# Targeting C-*myc* G-Quadruplex: Dual Recognition by Aminosugar-Bisbenzimidazoles with Varying Linker Lengths

**DOI:** 10.3390/molecules181114228

**Published:** 2013-11-18

**Authors:** Nihar Ranjan, Dev P. Arya

**Affiliations:** Laboratory of Medicinal Chemistry, Department of Chemistry, Clemson University, Clemson, SC 29634, USA; E-Mail: nranjan@g.clemson.edu

**Keywords:** G-quadruplex, neomycin, Hoechst 33258, oncogenes, FID, C-*myc*

## Abstract

G-quadruplexes are therapeutically important biological targets. In this report, we present biophysical studies of neomycin-Hoechst 33258 conjugates binding to a G-quadruplex derived from the C-*myc* promoter sequence. Our studies indicate that conjugation of neomycin to a G-quadruplex binder, Hoechst 33258, enhances its binding. The enhancement in G-quadruplex binding of these conjugates varies with the length and composition of the linkers joining the neomycin and Hoechst 33258 units.

## 1. Introduction

**Figure 1:**
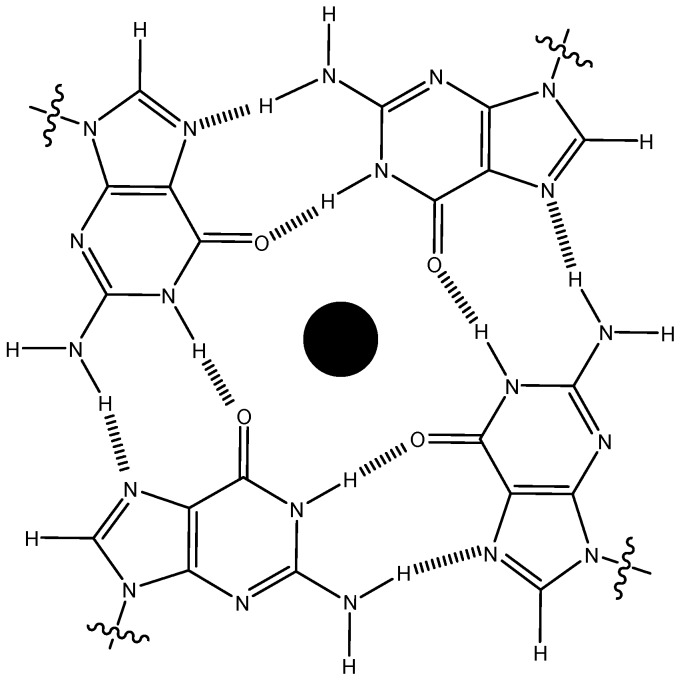
The arrangement of guanine bases in a G-tetrad. The cavity in the middle is occupied by the metal cations.

Human genomic DNA contains contiguous stretches of repetitive guanine bases that may fold into higher order DNA structures such as G-quadruplexes [1,2]. For example, many promoter sequences, as found in *C-myc*, VEGF, and *bcl-2*, *C-kit* have been observed to fold into four stranded G-quadruplex structures in solution [3]. G-quadruplex formation has been suggested to play important roles in cancer regulation [4,5,6,7] and has been a subject of intense investigation in recent times. Chromosomal ends, which are guanine rich, are often referred to as telomeres and have been shown to form G-quadruplex structures [8]. Understanding of G-quadruplex formation by telomeres, and its subsequent role in cellular functions, is vital for developing chemotherapeutic agents for treating cancer. There has been substantial *in vitro* evidence showing that G-quadruplex nucleic acid structures may be important for controlling the function of an enzyme called telomerase, which is critical to the growth of tumor cells [9]. Some recent reports have demonstrated detection of G-quadruplexes in human cells [10,11,12]. A number of G-quadruplex-binding small molecules have been reported in the last decade [13]. A common feature of these G-quadruplex-binding molecules is the presence of an extended aromatic ring system that allows binding through π-π overlap of G-tetrads (Figure 1) [14]. Some of these G-quadruplex-binders include porphyrin derivatives [15], oxazoles [16], and perylene derivatives [17,18] and similar systems that have fused π-ring systems within the molecule [19,20,21,22,23]. In addition, high affinity sequence-specific B-DNA minor groove binders have also been shown to interact with G-quadruplexes (although with weaker binding than to B-DNA) [24]. Non-planar molecules that interact with G-quadruplexes are very rare. We have recently found that a completely non-planar molecule neomycin, an aminoglycoside, interacts with certain G-quadruplex structures with moderate [25] to high affinities (unpublished results). Modeling and initial NMR structural studies have shown that the G-quadruplex grooves are the binding sites for neomycin [25]. Interestingly, aminoglycosides have also been reported to inhibit telomerase function [26]. 

**Figure 2:**
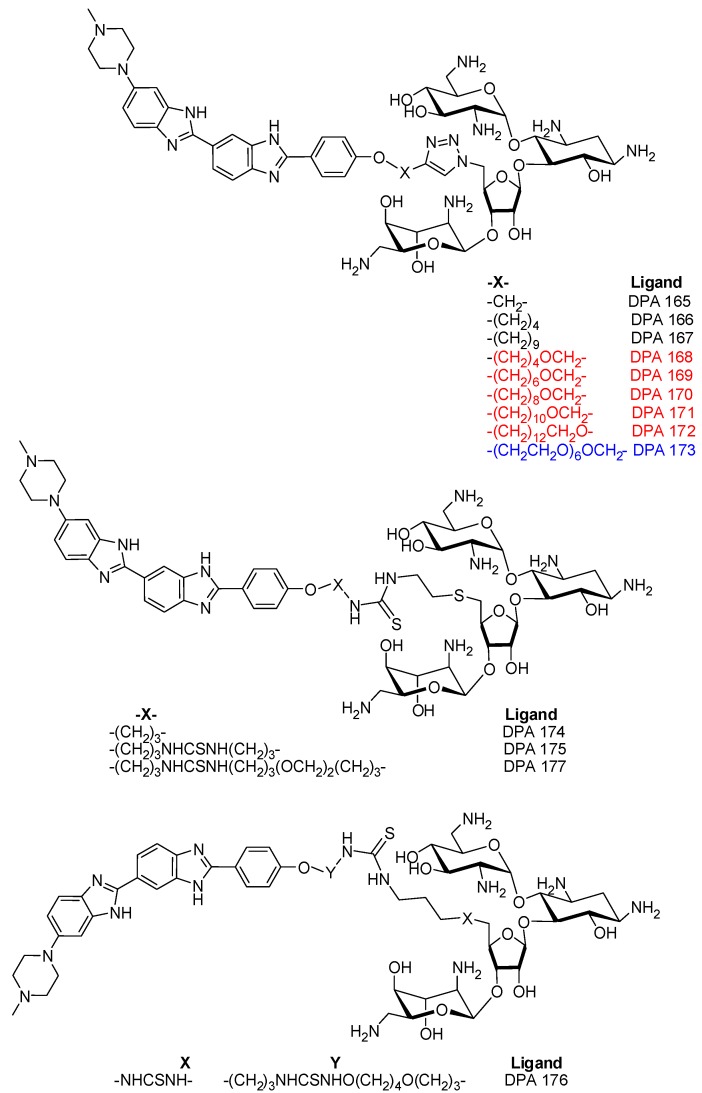
Structures of neomycin-Hoechst 33258 conjugates used in the studies.

In our previous studies we have shown that conjugation of select nucleic acid binders results in higher affinity [27] of the conjugate for a variety of nucleic acid structures [28]. Our approach has included conjugation of a major groove binder neomycin to a variety of other types of nucleic acid binders (major groove, [29] minor groove [30] or intercalators [31]). These studies have shown that covalent attachment of the two binding moieties results in a higher affinity binder, and that their binding is affected by the length of the linker joining the two units [30,32]. Extending dual recognition by aminoglycoside derivatives to G-quadruplexes, we have recently shown that by covalently attaching neomycin to a perylenediimide unit, peryelene binding to the human telomeric DNA can be improved [31]. Similarly, a neomycin-anthraquinone conjugate has displayed a nearly 1,000-fold increase in binding affinity to human telomeric G-quadruplex over neomycin [33]. A recent study has also reported that aminoglycosylation enhances G-quadruplex binding of epigallocatechin [34]. In this report, we explore the binding of two nucleic acid binders, neomycin and Hoechst 33258, conjugated by a covalent linkage, to unimolecular G-quadruplexes formed by a well-studied promoter sequence from the C-*myc* gene. Both of these nucleic acid binders have been shown to interact with moderate affinities to certain G-quadruplexes. Hoechst 33258 has been reported to bind with micromolar affinities to C-*myc* quadruplex in the presence of potassium ions [24]. Conjugation of neomycin, a G-quadruplex binder with groove binding as potential mode interaction, to Hoechst 33258 was thus envisaged to lead to enhanced binding towards C-*myc* quadruplex through simultaneous groove binding and stacking interactions. A small library of molecules (Figure 2) that contains neomycin and Hoechst 33258 units connected by linkers of varying length and composition has been evaluated to test the effect of linkers on the binding of G-quadruplexes.

## 2. Results and Discussion

### 2.1. Selection of the G-Quadruplex

Transcriptional promoter sequences are G-rich and can potentially form G-quadruplexes. Evidence suggesting the presence of G-quadruplexes in promoter sequences *in vivo* is continuously growing [35]. One of the earliest and most studied G-quadruplexes found in promoter sequences is the nuclear hypersensitive element (NHE-III_1_) of C-*myc* protooncogene, which forms a parallel-stranded G-quadruplex structure [36]. Since NHE-III_1_ region of C-*myc* (referred as C-*myc* quadruplex henceforth) forms a stable parallel structure in solution and contains wide pockets, we opted to study this quadruplex since our recent studies have indicated that wide groove-like domains found in *Oxytricha nova* and human telomeric DNA G-quadruplexes are the probable binding sites for neomycin [25]. Binding was compared to a biologically relevant duplex sequence that contains both purine and pyrimidine bases. The base sequences of the oligonucleotides studied are shown in Table 1.

**Table 1:** DNA base sequences of the oligonucleotides used in the study.

Abbreviation	DNA sequence (5ʹ-3ʹ)
C-*myc* quadruplex	TGGGGAGGGTGGGGAGGGTGGGGAAGG
TFO Active against C-*myc* duplex ^a^	AGGGAGGGAGGTAAGAAAAAGGG
TFO Active against C-*myc* 1 duplex ^a^	GGAAGGGGTGGGAGGGGTGGGAGGGG

^a^ TFO ‒ Triplex forming oligonucleotide.

### 2.2. Fluorescent Intercalator Displacement (FID) Assay

FID allows rapid screening of nucleic acid binding agents via a simple assay in which a large number of nucleic acid binders can be identified for their structure/sequence specificity [37,38]. This assay relies on the fluorescence response of an intercalator (usually thiazole orange (TO) or ethidium bromide). The intercalator is mixed with a nucleic acid sample in an appropriate ratio (which can be determined from a titration experiment), which results in a large increase in the fluorescence emission of the intercalator due to its binding to the nucleic acid. The intercalator can then be displaced by addition of the ligand of interest. The fluorescence emission changes corresponding to the ligand addition can be utilized to construct a binding isotherm. The binding isotherm can then be used to obtain information about the binding stoichiometry and association constant. In addition, the qualitative binding affinity and sequence specificity of a ligand can also be determined. Monchaud and coworkers have shown that this assay can also be utilized to identify G-quadruplex binders [39]. Using this FID assay we have identified duplex, triplex and quadruplex nucleic acid binders previously [25,29,40,41]. This method was applied in this study to screen neomycin-Hoechst 33258 conjugates DPA 165–177 for their binding to C-*myc* DNA quadruplex.

**Figure 3:**
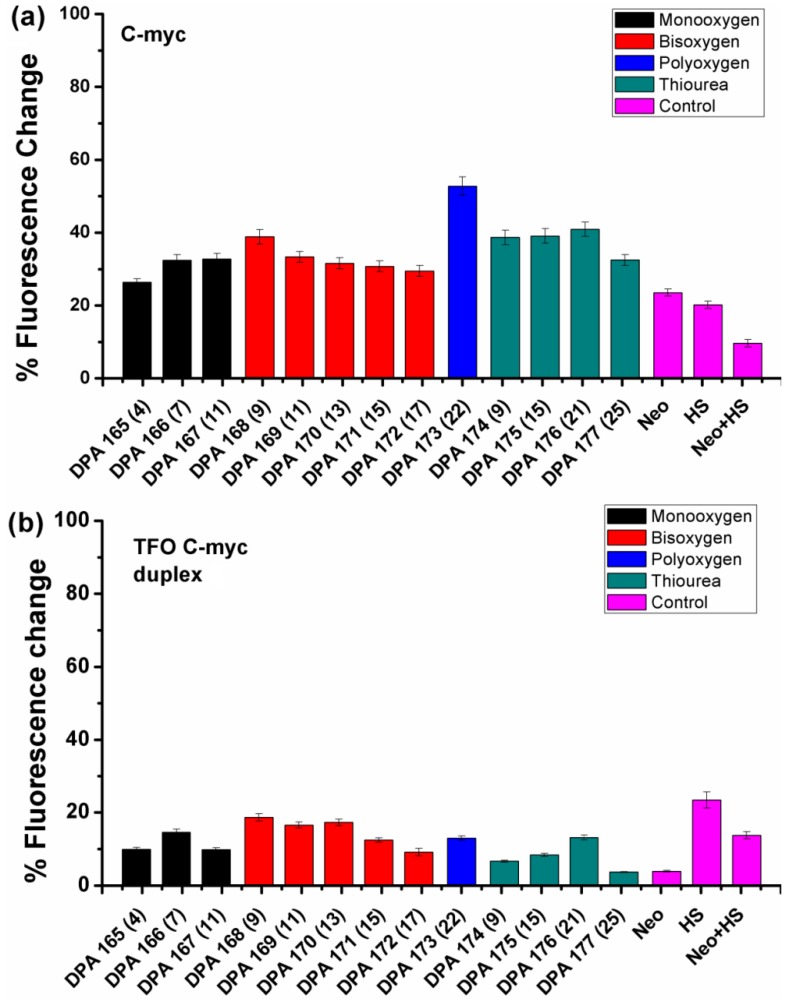
FID plots showing the change in the fluorescence upon ligand binding in a DNA -thiazole orange (TO) complex (**a**) C-*myc* quadruplex (**b**) TFO C-*myc* duplex. The number of atoms between the neomycin and Hoechst 33258 units in these conjugates is displayed in the parenthesis on the X-axis. Neomycin and Hoechst 33258 have been abbreviated as ‘Neo’ and ‘HS’ in the figure.

A fluorescence titration experiment was initially performed to determine the ratio of thiazole orange to *C-myc* quadruplex needed for the FID experiment. The titration revealed binding of two TO molecules per C-*myc* quadruplex at the saturation point (Figure S1, Supplementary Materials). Thus, a 1:2 complex of C-*myc* quadruplex:TO was utilized for subsequent studies. As displayed in Figure 3, a comparison of the FID displacement plots of DPA 165–DPA 177 binding to C-*myc* quadruplex and a DNA duplex is shown. A few notable trends can be immediately discerned. First of all, the addition of neomycin-Hoechst 33258 conjugates DPA 165–DPA 177 in C-*myc* quadruplex results in much better displacement of the bound intercalator than neomycin or Hoechst 33258 alone (Figure 3a). This shows that the covalent conjugation of neomycin and Hoechst 33258 that leads to conjugates DPA 165–DPA 177 (Figure 2), resulted in a ligand that binds more tightly to the C-*myc* quadruplex, as compared with the unconjugated analogues. The enhanced binding of conjugates DPA 165–DPA 177 can likely be attributed to the dual binding of neomy*c*in and Hoechst 33258 moieties at independent sites. As mentioned before, neomycin has been suggested to bind in the wide groove of *Oxytricha nova* and human telomeric quadruplexes. A similar interaction of neomycin within the C-*myc* quadruplex grooves is likely in this case as well. Secondly, there is a dependence of ligand (DPA 165–DPA 177) binding to the amount of thiazole orange displaced with respect to linker composition. As seen in Figure 3a, ligands that had linkers containing a single oxygen atom (DPA 165–DPA 167, black bars) showed increased displacement of TO from the quadruplex with increasing atom spacing (4–11 atoms). The ligands that contained bis oxygen linkers (DPA 168–DPA 172, red bars) showed decreased displacement as the atom spacing increased from 9 to 11 but further increase in the atom spacing (up to 17 atoms) showed very small changes in the displacement of TO, whereas the only ligand that had a polyoxygen atom in its linker (DPA 173, blue bar) showed the best displacement among all. The ligands that had linkers containing thiourea units (DPA 174–DPA 177) showed small increase in TO displacement as atom spacing increased from nine to 21 which was followed by decreased displacement of TO as atom spacing increased to 25 (green bars). Clearly the differences in the linker structure itself cause differential binding. This is likely because of the differing lengths, orientations and hydrogen bond donor/acceptor interactions for each linker and the subsequent binding impact with the quadruplex grooves and loops.

To compare the binding of the conjugates DPA 165–DPA 177 between *C-myc* quadruplex and duplex DNA structures, we performed FID experiments with a duplex DNA structure (designated TFO C-*myc* duplex) formed by adding the complementary DNA sequences to the sequences represented by TFO *C-myc* duplex. The CD spectrum of TFO *C-myc* duplex shows a broad positive CD signal in the region of 270–280 nm, which suggests possible presence of both A-form and B-form features in this duplex. The duplex formation was further checked by thermal denaturation experiment and analysis of the thermal difference spectrum (Figures S2 and S3 Supplementary Materials) [42].

We then compared the duplex DNA binding of the conjugates DPA 165–DPA 177 with the *C-myc* quadruplex-binding. As shown in Figure 3b, the FID displacement plots for the conjugates DPA 165-DPA 177 are significantly different than the FID displacement profile of the quadruplex. While the quadruplex showed the best displacement with the conjugate having large atom spacing (DPA 173), the duplex DNA showed higher or closely comparable displacement of neomycin-Hoechst 33258 conjugates with short atom spacing in most of the cases. Moreover the percent fluorescence change for quadruplexes (26%–52%) is much higher than the same for duplexes (~20% or less). These results show the overall duplex *versus* quadruplex selectivity for different linkers of the neomycin-Hoechst 33258 conjugates. Additionally, the percent fluorescence change (with duplex) for conjugates DPA 165–DPA 177 as compared to Hoechst 33258 alone is lower in contrast to the change observed for their binding to C-*myc* quadruplex binding. In fact, for TFO *C-myc* duplex, Hoechst 33258 caused the largest change in the fluorescence. We also performed FID displacement experiment with another DNA sequence (Figure S4, Supplementary Materials) designated as TFO C-*myc* 1 duplex (Table 1) which, as suggested by their thermal difference spectra [42], possibly exists as a mixture of duplex and triplex structures (Figure S3, Supplementary Materials). In this case also, we saw differential displacement of TO from the DNA-TO complex. Though, the conjugates showed lower displacement of TO from this duplex in comparison to the C-*myc* quadruplex, the displacement was slightly enhanced in comparison to TFO C-*myc* duplex which could be likely because of neomycin’s preference for triplexes [43,44]. However, it must be borne in mind that this data interpretation assumes that the displaced probe (TO) and the binding ligand compete for the same binding site in these structures and is purely qualitative in nature. Lack of displacement of TO by a ligand does not rule out the binding of the ligand, as the ligand could be binding to a different site that may not cause enough allosteric changes to weaken TO binding.

We then decided to study the interaction of DPA 173 (which afforded the highest displacement of thiazole orange) *C-myc* quadruplex binding for further biophysical studies. The quadruplex *versus* duplex selectivity of DPA 173 was quantitated using fluorescent intercalator displacement titration. The serial addition of DPA 173 to the nucleic acid-TO complex led to quenching of the fluorescence emission of thiazole orange (Figure S5, Supplementary Materials). After complete displacement of the thiazole orange (which is indicated by negligible changes in the fluorescence emission intensity of thiazole orange), DC_50_ values can be determined (DC_50_ refers to amount of ligand quadruplex required to displace 50% of the bound intercalator). The DC_50_ values obtained from FID titrations are listed in Table 2. As reflected from the DC_50_ values, there is a distinct preference of DPA 173 for C-*myc* quadruplex in comparison to duplex structures. The DC_50_ value for C-*myc* quadruplex was 0.48 µM while for TFO C-*myc* duplex and TFO C-*myc* 1 it was 1.07 and 1.22 µM respectively. The smaller DC_50_ value for quadruplex in comparison to duplexes shows the preference of DPA 173 for C-*myc* quadruplex. Control experiments with Hoechst 33258 and neomycin were also performed which also supported that the conjugated neomycin and Hoechst 33258 units are much better C-*myc* quadruplex binders than neomycin or Hoechst 33258 itself.

**Table 2:** DC_50_ values obtained by titration of various nucleic acids using DPA 173.

Nucleic Acid	Ligand	DC_50_ (µM)
C-*myc*	DPA 173	0.48 ± 0.04
C-*myc*	Hoechst 33258	1.58 ± 0.01
C-*myc*	Neomycin	>5.5
C-*myc* duplex	DPA 173	1.07 ± 0.09
C-*myc* duplex 1	DPA 173	1.22 ± 0.17

### 2.3. Circular Dichroism Studies of DPA 173 and Hoechst 33258 Binding to C-myc G-Quadruplex

**Figure 4:**
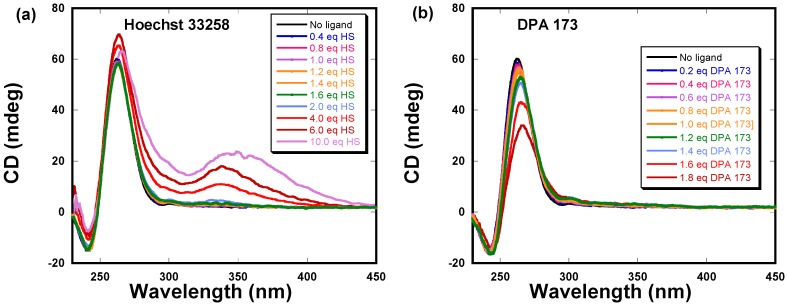
Circular dichroism studies of *C-myc* quadruplex with (**a**) Hoechst 33258 and (**b**) DPA 173.

We utilized circular dichroism to further characterize the binding of the DPA 173-*C-myc* quadruplex interaction. As shown in Figure 4, the CD spectrum of *C-myc* shows a positive band at 260 nm, which is characteristic of the formation of a parallel quadruplex. The G-quadruplex was then titrated with both Hoechst 33258 and DPA 173 respectively. As shown in Figure 4a, incremental addition of Hoechst 33258 to the quadruplex resulted in small changes in the positive band at 260 nm. However, induced CD signals were observed at 339 nm, which corresponds to the chromophore (Hoechst 33258) absorption wavelength. The appearance of induced CD as well as the changes in the positive intensity at 260 nm suggests the binding of Hoechst 33258 to this quadruplex. Previous studies have also characterized the binding of Hoechst 33258 to this C-*myc* quadruplex by spectroscopic methods [24]. However, we observed the discernible induced CD signals only at ratios above two Hoechst 33258:quadruplex. This observation suggests the requirement of high ligand concentration for the induced CD to be observed. In contrast to the Hoechst 33258 binding, the binding of DPA 173 resulted in much larger changes at the CD band at 260 nm. However, we did not observe any induced signal in the Hoechst 33258 absorption region of DPA 173. 

As shown in Figure 4a for Hoechst 33258 complexation, the induced CD spectrum was observed only above a ligand:quadruplex ratio of two. In the case of DPA 173, we could not study the DPA173-*C-myc* interaction at ligand:quadruplex ratio over two, as sample precipitation was observed in the nucleic acid-ligand complex solution when the ligand:quadruplex ratio exceeded 1.8.

### 2.4. UV-Vis Absorption Studies

**Figure 5:**
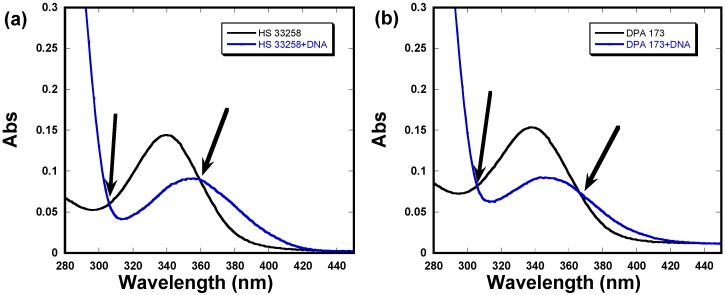
UV-Vis absorption spectra of (**a**) Hoechst 33258 and (**b**) DPA 173 in the presence of C-*myc* quadruplex. Arrows indicated isobestic points.

UV-Vis absorption studies were also performed to look into to binding of DPA 173 to C-*myc* quadruplex and a comparison experiment was also performed with Hoechst 33258. In the absence of C-*myc* quadruplex, Hoechst 33258 shows absorption maximum at 339 nm (Figure 5a). 

Upon addition of Hoechst 33258, hypochrosim was observed which was accompanied by a 19 nm bathochromic shift in the absorption maximum. Similar changes were observed in the case of DPA 173 (Figure 5b) both in the hypochrosim and bathochromic shift (10 nm). Such hypochroism is indicative of ligand interaction through stacking as has been observed before [24]. Both Hoechst 3358 and DPA 173 also display isobestic points (indicated by arrows in Figure 5) which suggest the complex formation. 

## 3. Experimental

### 3.1. Synthesis of DPA 165-DPA177

Synthesis of compounds DPA 165-DPA177 was performed in-house and details will be published elsewhere.

### 3.2. Nucleic Acids

All DNA oligonucleotides were purchased from Eurofins MWG Operon (Huntsville, AL, USA) and used without further purification. The nucleic acid concentrations were determined using extinction coefficients provided by the supplier. Nucleic acid solutions were prepared in buffer 10 mM Tris-HCl, 0.1 mM EDTA and 100 mM KCl at pH 7.5. The nucleic acid solutions were prepared by heating at 95 °C for 15 min, and then slowly cooling back to room temperature. After incubation for two weeks, the quadruplex formation was checked by CD spectroscopy. The stock solution was stored at 4 °C and diluted to desired concentrations as required.

### 3.3. Fluorescent Intercalator Displacement (FID) Experiments

Fluorescence experiments were performed on a Tecan Genois (Mannedorf, Switzerland) fluorimeter equipped with a 96 well plate reader. All experiments were performed at room temperature (21–23 °C). The experiments were performed in the 96 well plates as triplicates. The DNA solution was prepared at 1 µM/quadruplex concentration in 10 mM Tris-HCl, 0.1 mM EDTA and 100 mM KCl at pH 7.5. The nucleic acid (0.25 µM/quadruplex or duplex) was then mixed with thiazole orange (TO) at a concentration of 0.50 µM. The ligand was added to the DNA-TO complex solution at 1:1 ratio and followed by a five minute equilibration time before the fluorescence emission spectrum was recorded (λ_Excitation_ = 485 nm and λ_Emission_ = 534 nm). The change in the fluorescence emission was plotted as:

% fluorescence change = (∆F/I_F_) × 100

where, ∆F = Change in fluorescence upon ligand addition and I_F_ = Initial fluorescence of the DNA-TO complex.

### 3.4. Circular Dichroism (CD) Spectroscopy

CD experiments were performed in a 3.0 mL quartz cell on a Jasco J-810 spectropolarimeter (Easton, MD, USA) equipped with a thermo-electrically controlled cell holder. The experiments were performed at 10 µM/strand concentration in buffer 10 mM Tris-HCl, 0.1 mM EDTA and 100 mM KCl at pH 7.5. The ligands were prepared as concentrated stock solutions and were added to DNA quadruplex solution serially in small aliquots (~1 µL). After each addition the solution was equilibrated for five minutes before the CD scan was taken. Each CD spectrum is an average of three scans. All experiments were performed at 20 °C.

### 3.5. Fluorescence Titration Based FID Experiments

Fluorescence titration experiments were conducted on a Photon Technology International (Lawrenceville, NJ, USA) instrument equipped with a temperature controller. The ligand solution (0.25 µM) was added with TO solution (0.25 µM). The DNA-TO complex was excited at 501 nm and the emission spectrum was recorded between 510–750 nm. Ligand of interest was added successively in small aliquots (1–2 µL) until no more changes in the fluorescence emission spectrum of the ligand were observed. The resulting emission profile was then normalized and plotted as % fluorescence change with respect to concentration. The point where 50% displacement of TO was observed was assigned as DC_50_. The experiments were performed in buffer 10 Tris-HCl, 0.1 mM EDTA and 100 mM KCl at pH 7.5.

### 3.6. UV-Vis Experiments

Ultraviolet (UV) spectra were collected on a Varian (Walnut Creek, CA, USA) Cary 100 Bio UV-Vis spectrophotometer equipped with a thermoelectrically controlled 12-cell holder. The experiments were performed at room temperature (22–24 °C) in quartz cells with 1cm path length. The DNA concentration was 5 µM/strand and the ligand concentration was 5 µM in each experiment. The buffer used in the experiments was 10 Tris-HCl, 0.1 mM EDTA and 100 mM KCl at pH 7.5.

## 4. Conclusions

G-quadruplex nucleic acids are therapeutically important targets for treating some of the fatal ailments of current times. A number of G-quadruplex binding ligands have been discovered in the past decade that has helped in our growing understanding of G-quadruplex targeting. Majority of these binders have micromolar affinities to the quadruplexes. Unlike more defined duplex and triplex nucleic acid structures, G-quadruplex structures have been found to have multiple folding patterns which leads to wide structural diversity within the G-quadruplexes itself. The majority of G-quadruplex binding ligands are planar and they bind with micromolar affinities. In an effort to enhance the binding of G-quadruplex binders, we have recently used the aminoglycoside conjugation to increase the G-quadruplex binding of ligands [33]. Our studies show that neomycin-Hoechst 33258 conjugates DPA 165–DPA 177 show stronger binding to C-*myc* quadruplex than do their constituent units Hoechst 33258 or neomycin. Comparison of the FID profiles of *C-myc* quadruplex to DNA duplexes show that the conjugates were able to better displace the thiazole orange bound to the quadruplex than the duplexes. In the present case, though the duplex to quadruplex selectivity is not very high, this study does highlight the role of aminosugars in the enhancement of G-quadruplex affinity. Possibly a more suitable combination of the G-quadruplex binder and the aminosugar could lead to a higher increase in the quadruplex to duplex selectivity. UV-Vis absorption studies indicated that Hoechst 33258 moiety of the conjugate makes stacking interactions with the quadruplex. Such interactions are likely occur on the surface of the tetrad facilitated by AT base containing loop regions in the quadruplex, as also suggested in a previous study [24]. Since neomycin lacks any planar unit, we propose its binding in the groove regions. FID experiments also showed that the binding is affected by the linker composition and length. Further studies in understanding the roles of linkers and linked moieties in target selectivity could aid in the future design of more effective and selective G-quadruplex-binding ligands.

## Supplementary Materials

Supplementary materials can be accessed at: http://www.mdpi.com/1420-3049/18/11/14228/s1.
