# Supplementary material for: Targeting C-myc G-Quadruplex: Dual Recognition by Aminosugar-Bisbenzimidazoles with Varying Linker Lengths
Source: Molecules. 2013 Nov 18;18(11):14228–40. doi: 10.3390/molecules181114228 (PMC6270413; doi:10.3390/molecules181114228)

Supplementary Materials

Figure S1. (**a**) Fluorescence titration of thiazole orange with *C-myc* quadruplex in buffer (10 mM Tris-HCl, 1.0 mM EDTA and 100 mM KCl at pH 7.5). Thiazole orange (78 nmol) was titrated with *C-myc* DNA quadruplex. A concentrated DNA solution was added in small aliquots (1–2 μL) followed by mixing with a magnetic stirrer and three minute equilibration before a scan was taken. Each scan represents an average of three readings. (**b**) Thiazole orange (TO) was excited at 502 nm and the emission spectrum was recorded between 510–700 nm.

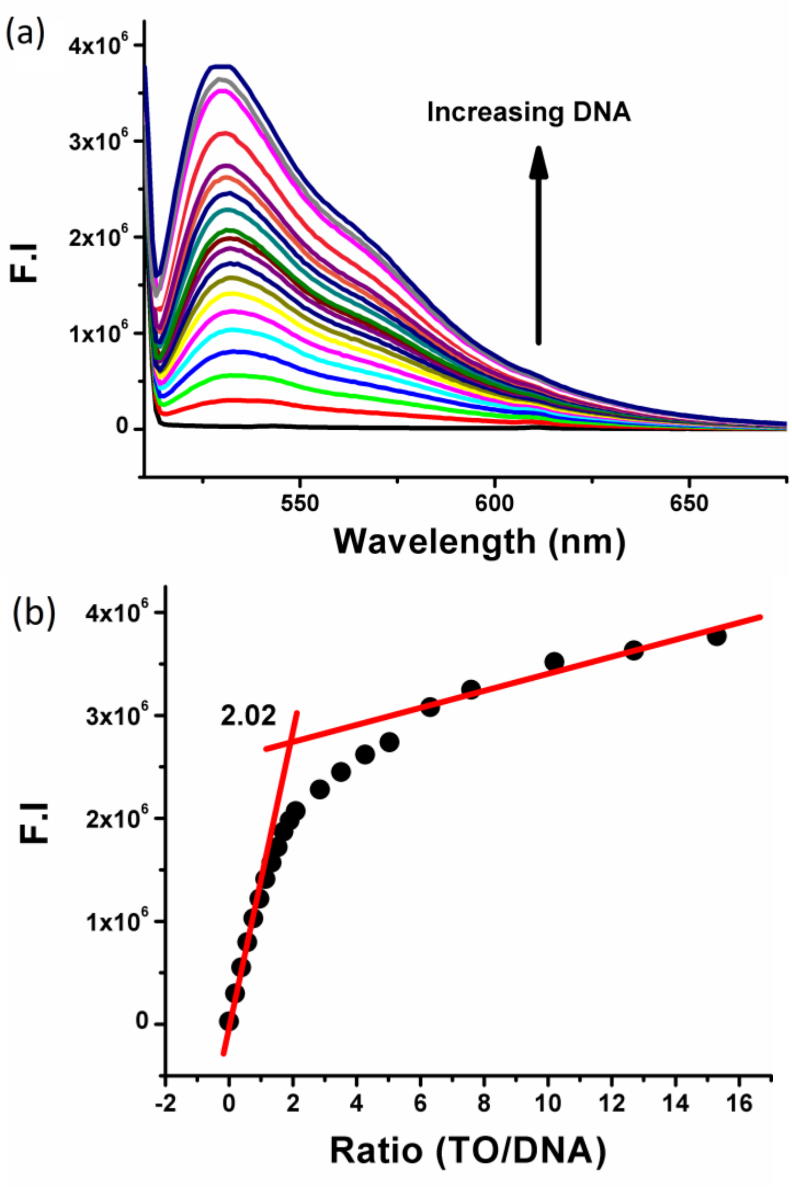

Figure S2. (**a**) CD spectrum of TFO *C-myc*1 duplex (**b**) TFO *C-myc* duplex (**c**) UV-thermal denaturation profile of TFO *C-myc*1 duplex (**d**) UV-thermal denaturation profile of TFO *C-myc* duplex. All experiments were performed in buffer 10 mM Tris-HCl, 0.1 mM EDTA and 100 mM KCl at pH 7.5. For CD experiments, the concentrations of the nucleic acids were 10 μM/duplex; for UV thermal denaturation experiments DNA concentrations were 2 μM/duplex. Each CD spectrum is an average of two scans. The CD experiments were conducted at room temperature (20–22 °C). The melting points were determined from a single experiment.

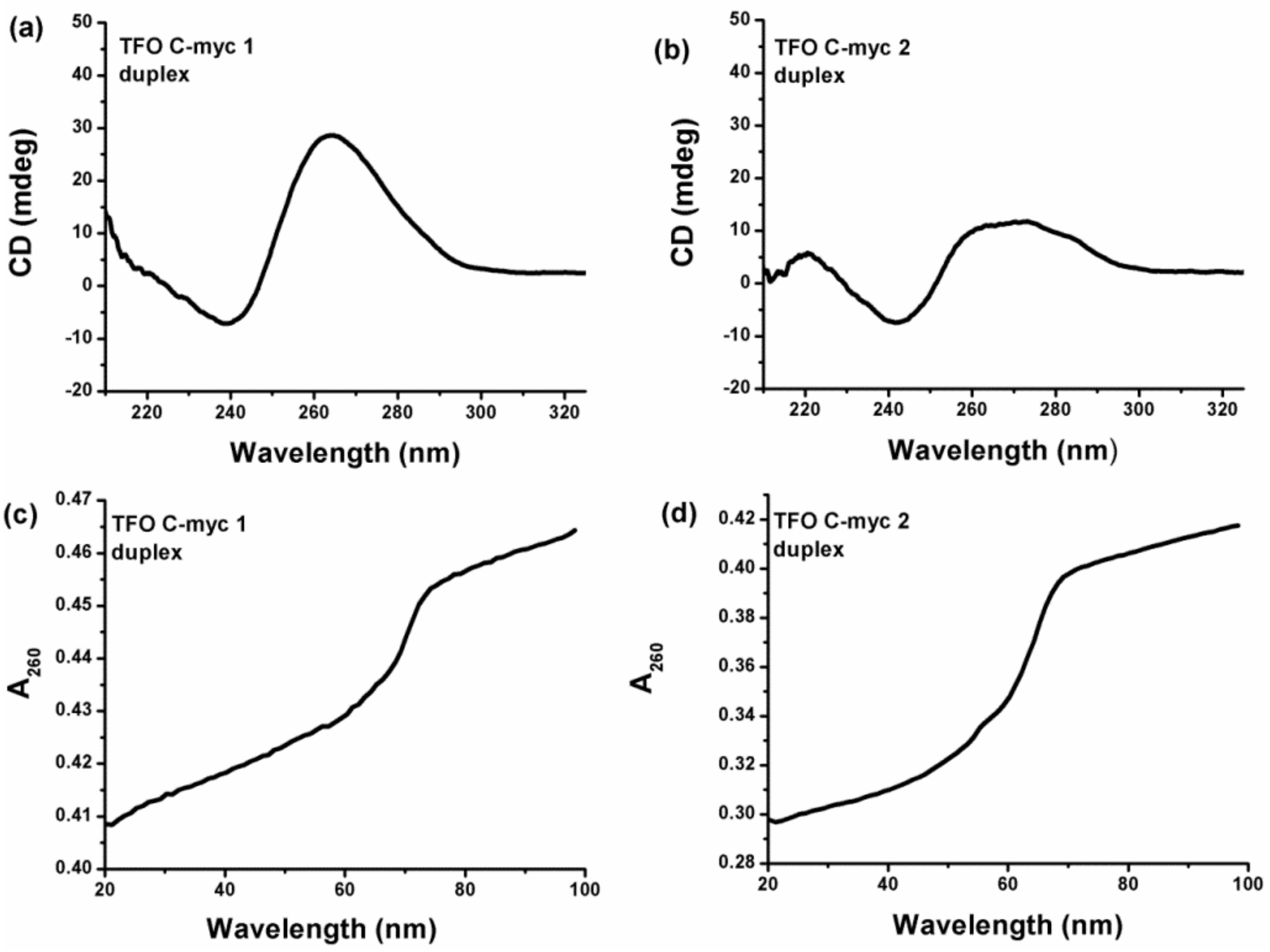

Figure S3. Thermal difference spectra of (**a**) TFO C-myc duplex and (**b**) TFO C-myc duplex 1. The experiments were performed at 1 μM/ duplex concentration in buffer 10 mM Tris-HCl, 0.1 mM EDTA, 100 mM KCl at pH 7.5. The absorption spectra of folded and unfolded forms of the duplexes were recorded at 20 °C and 85 °C respectively.

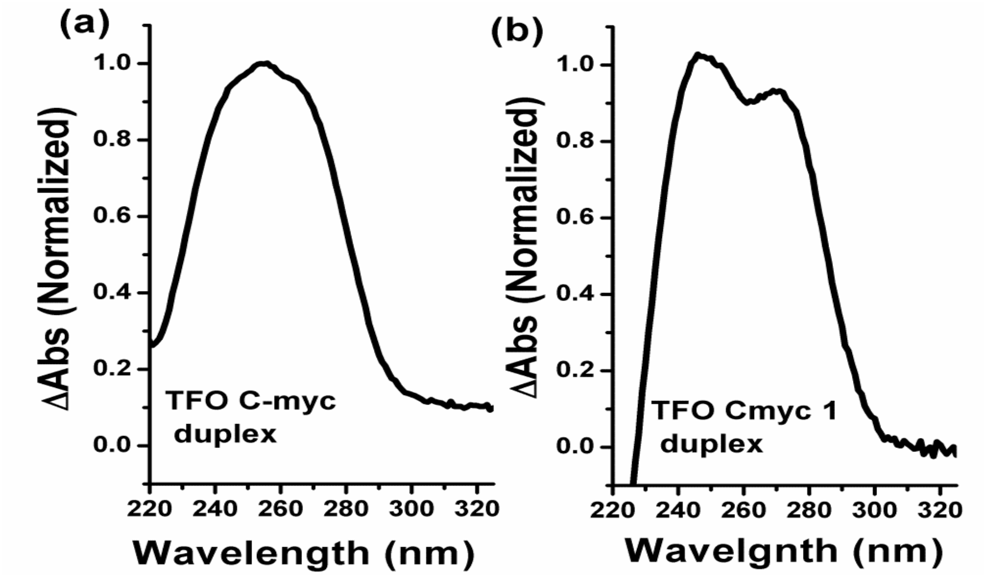

Figure S4. FID plot showing the percent change in the fluorescence emission upon ligand binding in C-*myc* -thiazole orange (TO) complex. [DNA] = 1 μM/duplex, [TO] = 2 μM, [Ligand] = 1 μM. All experiments were performed in buffer 10 mM Tris-HCl, 0.1 mM EDTA and 100 mM KCl at pH 7.5.

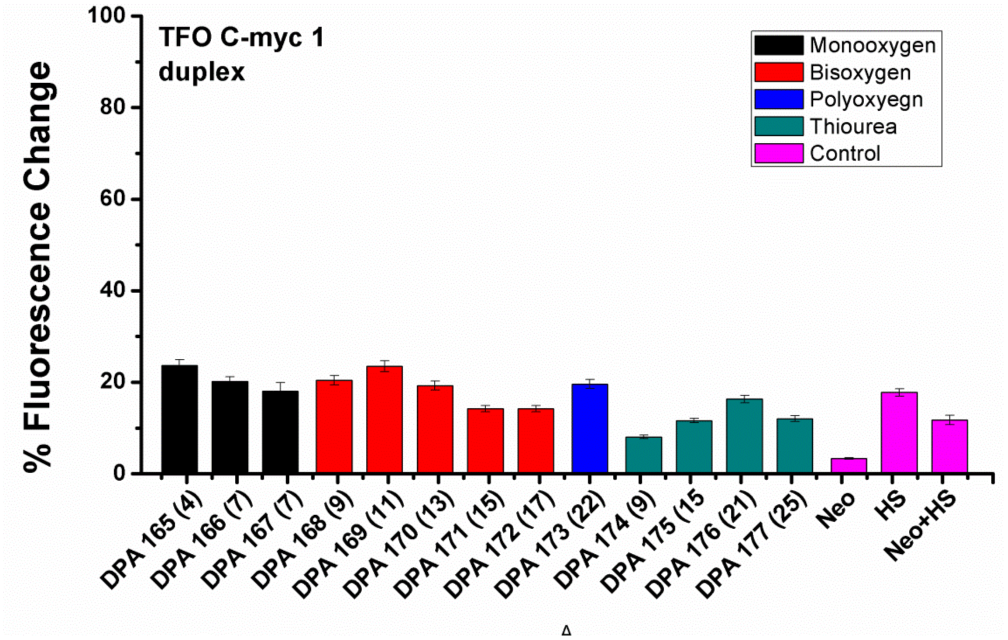

Δ

Figure S5. FID titration profile of (**a**) DPA 173 being titrated into C-*myc* quadruplex (**b**) Hoechst 33258 being titrated into C-*myc* quadruplex (**c**) neomycin being titrated into C-*myc* quadruplex and (**d**) DPA 173 being titrated into TFO C-*myc* duplex (**e**) DPA 173 being titrated into TFO C-*myc* 1 duplex. The DNA (0.25 μM) was mixed with thiazole orange (0.50 μM) followed by successive addition ligands at different ratios as indicated on the graph. Each spectrum is an average of two scans. Thiazole orange was excited at 501 nm and the emission was recorded between 510–750 nm. The experiments were performed in buffer 10 mM tris-HCl, 1.0 mM EDTA and 100 mM KCl at pH 7.5.

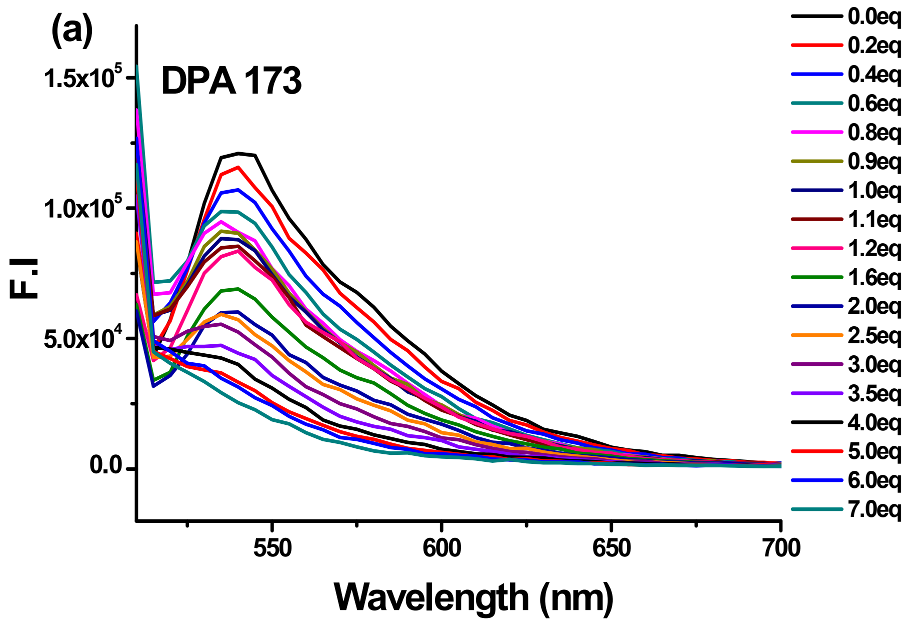

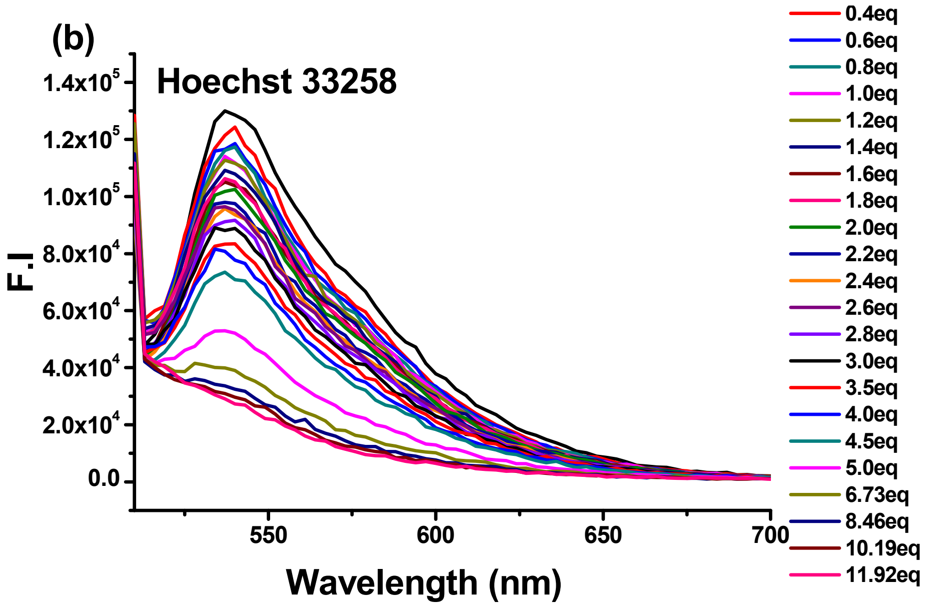

Figure S5. *Cont.*

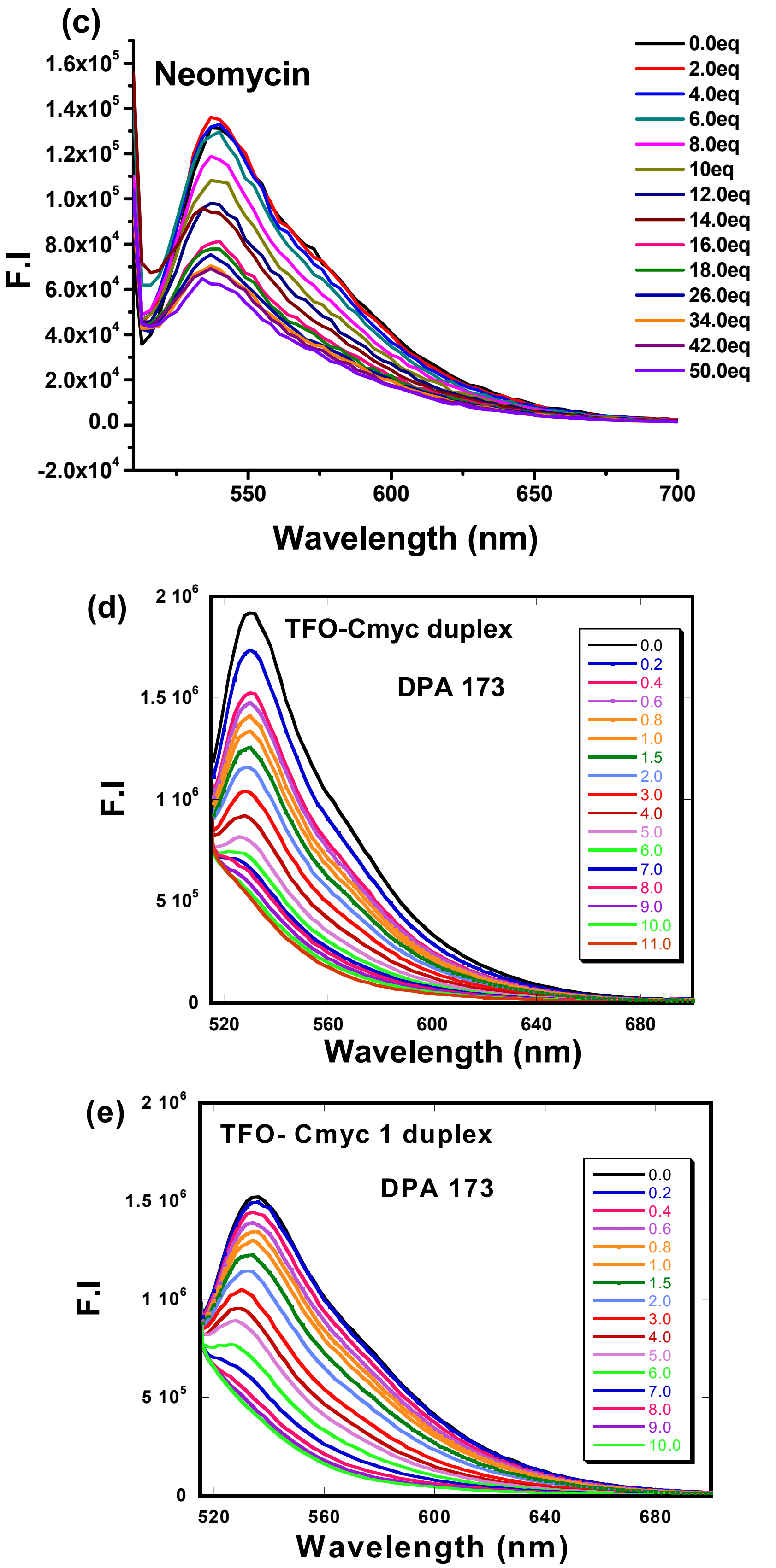

Supplement: Supplementary file 1 [file molecules-18-14228-s001.pdf]
